# Suicidality and self-harm in adolescents before and after the COVID-19 pandemic: a systematic review

**DOI:** 10.3389/fpsyt.2025.1643145

**Published:** 2025-09-19

**Authors:** Danilo Bastos Bispo Ferreira, Renata Maria Silva Santos, Maria Carolina Lobato Machado, Victhor Hugo Martins Rezende, Patrícia Gazire de Marco, Marco Aurélio Romano-Silva, Débora Marques de Miranda

**Affiliations:** ^1^ Department of Pediatrics, Federal University of Minas Gerais, Belo Horizonte, Brazil; ^2^ Department of Psychiatry, Federal University of Minas Gerais, Belo Horizonte, Brazil

**Keywords:** adolescents, suicidal behavior, suicidality, suicide attempt, self-harm, COVID-19 pandemic, mental health, risk factors

## Abstract

**Introduction:**

Adolescent mental health, self-harm, and suicidality are critical concerns during this developmental stage, marked by intense physical, emotional, and social changes. The COVID - 19 pandemic has further intensified these vulnerabilities by disrupting daily routines, increasing social isolation, limiting access to mental health services, and exacerbating academic and emotional stressors.

**Methods:**

This systematic review followed the PRISMA 2020 guidelines and employed the PECO strategy to identify relevant studies. A total of 55 quantitative studies published between 2010 and 2024 were included. These studies examined the prevalence and risk factors of self-harm and suicidal behaviors among adolescents aged 10 to 19 years, comparing findings from the pre-pandemic and pandemic periods. Psychosocial, economic, and cultural determinants were also evaluated.

**Results:**

The analysis revealed a consistent increase in self-harm and suicidality during the pandemic, with adolescent girls being disproportionately affected. Gender disparities were observed across diverse cultural contexts. Contributing factors included social isolation, excessive screen time, reduced access to education and healthcare, and increased family or financial stress. Cultural variability shaped both prevalence and clinical expression.

**Discussion:**

These findings underscore the amplifying effect of the COVID - 19 pandemic on adolescent mental health vulnerabilities and highlight the need for culturally sensitive, gender-informed preventive strategies. Public policies should prioritize mental health support for youth and address systemic inequities to mitigate the psychological consequences of global crises. This review offers important insights into adolescent mental health in times of collective adversity.

**Clinical trial registration:**

PROSPERO https://www.crd.york.ac.uk/prospero/display_record.php?ID=CRD42024538641, identifier CRD42024538641.

## 1 Introduction

Adolescence is a crucial transition period between childhood and adulthood, characterized by significant changes in physical, emotional, and social development. The World Health Organization (WHO) defines adolescence as the stage of life that spans from ages 10 to 19, a critical period during which profound biological and psychological transformations occur, alongside the emergence of new vulnerabilities, particularly related to mental health (1). Adolescents have an increased risk of self-harm and suicidal behaviors, especially in contexts of social and economic crises, such as the COVID - 19 pandemic (2).

The COVID - 19 pandemic brought a series of negative impacts on adolescent mental health, exacerbating pre-existing conditions and generating new psychological challenges (3). The prolonged closure of schools, social isolation, disruption of daily activities, and increased use of electronic devices contributed to the worsening of conditions such as depression, anxiety, self-harm, and suicidality (4, 5). Previous studies had already identified a growing trend of self-harm and suicidal behavior especially among girls before the pandemic; however, the pandemic period intensified these patterns (6, 7). Factors such as loneliness, bullying, family conflicts, and economic difficulties emerged as significant risk factors during the pandemic related to the increase in adolescents’ vulnerability to mental health crises. Gender disparities and cultural variations modulates the expression of suicide-related behaviors, suggesting that effective interventions must be sensitive to the specific needs of different population groups (8, 9).

In Latin America, estimates from the Global Burden of Disease 2019 show that while the absolute number of suicide deaths rose in most countries between 1990 and 2019, age-standardized rates varied substantially across settings; suicide burden is consistently higher among males, peaks in youth/early adulthood, and often tracks with sociodemographic development, underscoring heterogeneous risk profiles across the region (10).

This systematic review aims to explore the prevalence and the risk features of self-harm and suicidal behaviors among adolescents in the period before and during the COVID - 19 pandemic, as a particular stressful and uncertain timing with disorganized coping strategies, comparing the changes in these two contexts. Contributing to the understanding of the complex interactions between psychosocial, economic, and cultural factors that influence adolescent mental health, this article looks to provide insights into the formulation of public policies and interventions that can mitigate the effects of this global crisis on youth.

## 2 Method

A systematic review was conducted to address the following research question: {it}”Was there a change in the prevalence and risk factors of suicidality and self-harm among adolescents before and after the onset of the COVID - 19 pandemic up to the present day?”{/it} The review followed the PRISMA (Preferred Reporting Items for Systematic Reviews and Meta-Analyses) protocol (11) and was registered in PROSPERO under the ID CRD42024538641. To define the search strategy, preliminary searches were independently conducted by two reviewers in the databases, following the PECO strategy, as detailed in **Table 1**.

**Table 1:** Description of PECO strategy elements.

Component	Indicator
P: Population of interest	Adolescent
E: Exposure	Worldwide stressful condition related to pandemic or social measures to mitigate infection
C: Comparison	Studied before the COVID pandemic and after the COVID - 19 pandemic
O: Outcome	Change in the prevalence of self-harm, suicidal ideation, suicide attempt, suicide

Considering the WHO concept of adolescence, individuals aged between 10-19 years were considered as adolescents for inclusion. The official search was conducted on 07/05/2024, combining the descriptors “adolescents”, “suicidality”, “self-harm”, and “prevalence”. For the post-pandemic search, the descriptor “COVID-19” was added. The descriptors were combined using the AND operator in the following databases: PubMed, PsycNet, Embase, and Scopus. To ensure a sensitive search, the following eligibility criteria were employed: quantitative studies in community samples with populations aged 10 to 19 years (adolescents), published in English between 2010 and 2024. The time frame was established to encompass the period of technological expansion with increased adolescent engagement with the internet, allowing comparison with the COVID - 19 pandemic period, aiming to assess the magnitude of these two events concerning the study outcomes. The search syntaxes for each database are available in the **Supplementary Material** of this article.

Only peer-reviewed articles were included, and grey literature was excluded. Qualitative studies, reviews of any type, case reports, case series, and studies conducted in clinical samples were also excluded. To ensure greater specificity, the researchers excluded studies that did not address the associations between prevalence and risk factors for suicidality and self-harm. Titles and abstracts were first screened independently and in a blinded manner; potentially eligible records then underwent independent full-text assessment, with disagreements resolved by consensus or a third reviewer. For the factor’s synthesis, a deductive–inductive codebook guided categorization; a study contributed to a category only when the factor was analytically examined and reported as associated with suicidality/self-harm; descriptive mentions were not counted. The selection procedures were performed by at least two researchers, independently and in a blinded manner, using the AI platform Rayyan (12). The consensus was reached by a discussion between the three authors D, MC and RM on which articles would remain in the review.

### 2.1 Data extraction

A standardized data extraction table was created to extract the information: reference and study type, country of publication, sample characteristics, study objectives, prevalence of self-harm and suicidality, main associations with risk factors, and the Evidence Quality Score of each article included in the review. The studies were listed in the table in alphabetical order. To define the magnitude of the associations found, Cohen’s d test was extracted from the articles, which reflects the statistical power analysis for behavioral sciences (13). If this test was not used, others such as Odds Ratio (OR) and regression tests, which could correspondingly provide the magnitude of the association, would be extracted from the studies, and effect sizes were defined according to the values presented. An OR of 1.44 corresponds to a small effect size, 2.47 to a medium effect size, and a large effect size begins at 4.25. For regression tests, 0.10 corresponds to a small effect size, 0.30 to a medium effect size, and a large effect size starts at 0.50 (14).

Although effect size measures (e.g., Cohen’s d, odds ratios, and regression coefficients) were extracted when available to enhance interpretative value, a meta-analysis was not feasible due to substantial heterogeneity among the included studies. Variability in study design (e.g., cross-sectional versus longitudinal), outcome definitions (such as suicidal ideation, non-suicidal self-injury, or suicide attempts), population characteristics, and measurement tools hindered statistical comparability. Therefore, a rigorous narrative synthesis was conducted, in line with PRISMA 2020 recommendations for systematic reviews without meta-analyses.

### 2.2 Quality assessment

The quality of the studies was assessed using the Joanna Briggs Institute (JBI) Critical Appraisal Tool. This checklist-based instrument was chosen due to its rigorous evaluation criteria for the included studies and its recommendation in the literature for use in analytical cross-sectional and cohort observational studies that seek to collect data on risk or causality (15, 16). The tool evaluates the detailed description of the sample to ensure compatibility with the population of interest. The study should clearly describe the exposure measurement method and clarify any potential confounding factors that may influence the interpretation of the results. Additionally, measurements should be conducted using validated instruments, and the statistical method used should be the most appropriate (16).

The available guidelines for applying the JBI tool do not specify a cutoff point for determining whether a study is of “High”, “Moderate”, or “Low” quality. Given the nature of the review and the inclusion of studies, mostly with a cross-sectional design, the team placed greater emphasis on criteria assessing risks of selection bias, measurement bias, and confounding bias, specifically questions 1, 3, 4, 5, 6, and 7. For cohort studies, greater weight was given to questions 1, 3, 4, 5, 6, 7, 8, and 9. Using this strategy, the overall methodological quality of each study was classified as “High”, “Moderate”, or “Low” based on the percentage of “Yes” responses considering these “key” items.

Studies classified as “High” quality must have 100% positive responses (“Yes”) to these key items. If they received one or two uncertain or negative responses (“Uncertain” or “No”, respectively), the methodological quality of the study was rated as “Moderate”. If there were more than two uncertain or negative responses (“Uncertain” or “No”, respectively), the study’s methodological quality was rated as “Low” (17). The evaluation was conducted in pairs, and discrepancies were resolved through consensus with a third reviewer.

## 3 Results

**Figure 1:**
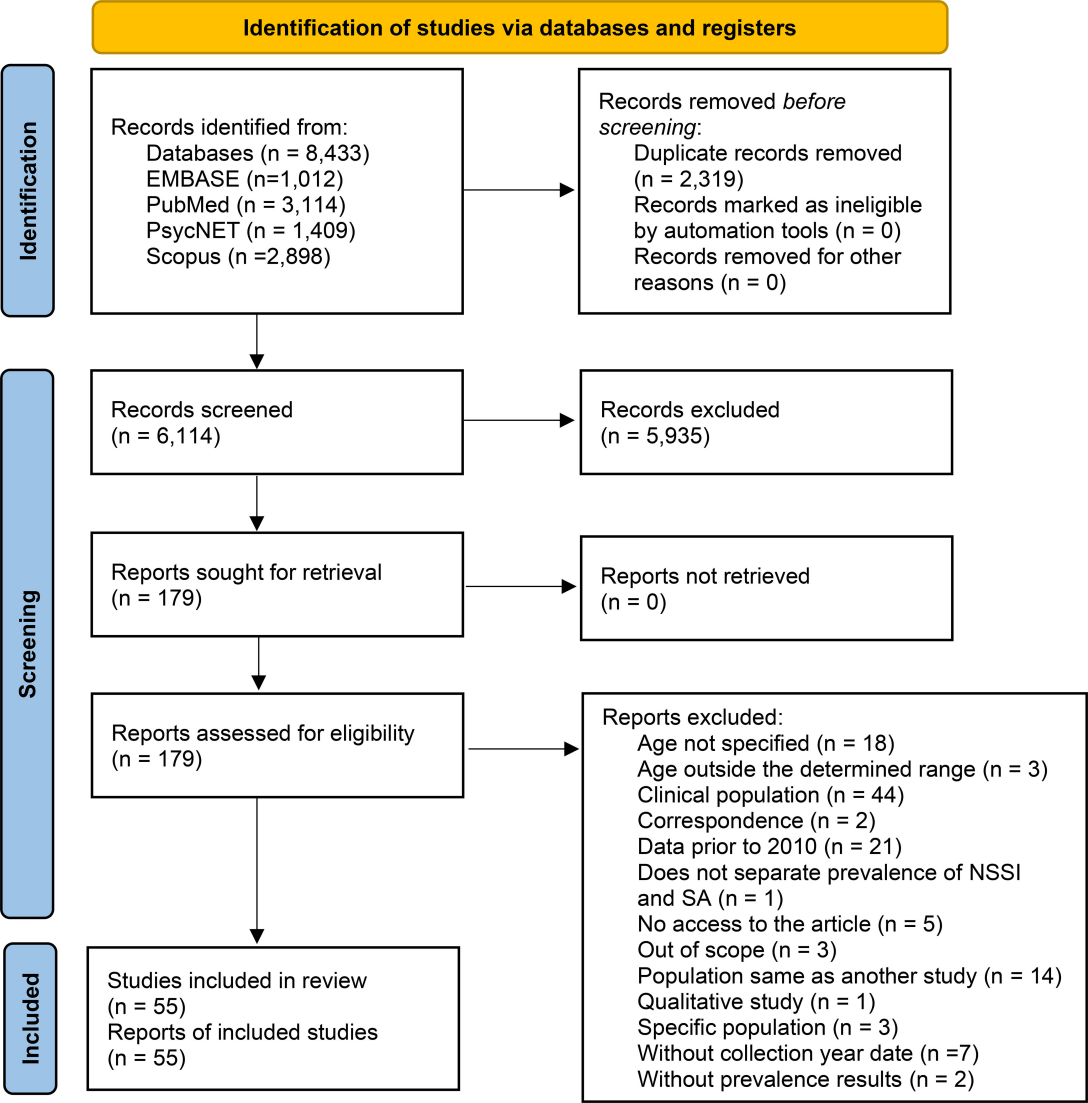
PRISMA flowchart.

The search yielded a total of 8,433 articles, of which 2,319 were duplicates, leaving 6,114 articles for title and abstract screening. At this stage, 5,935 articles were excluded for not meeting the pre-established inclusion criteria. Therefore, 179 articles were read in full, and 55 were included in this study, considering the inclusion of full-text articles retrieved. **Figure 1** - PRISMA flow study diagram (18), illustrates the selection process.

### 3.1 Study characteristics

A total of 55 articles on suicidal behavior published between 2010 and 2024 were included in this study. Of these, 45 were cross-sectional (27 from the pre-pandemic period and 18 from the post-pandemic period), and 10 were longitudinal (3 from the pre-pandemic period and 7 from the post-pandemic period). Among the 30 articles analyzing self-harm and suicidal ideation during the pre-pandemic period, only 1 addressed both topics comprehensively. The remaining studies focused on a single dimension: 4 exclusively on non-suicidal self-injury (NSSI) and 25 solely on suicidality. In contrast, of the 25 articles exploring the post-pandemic period, 3 examined both topics, while 7 focused on NSSI and 15 on suicidality.

**Figure 2:**
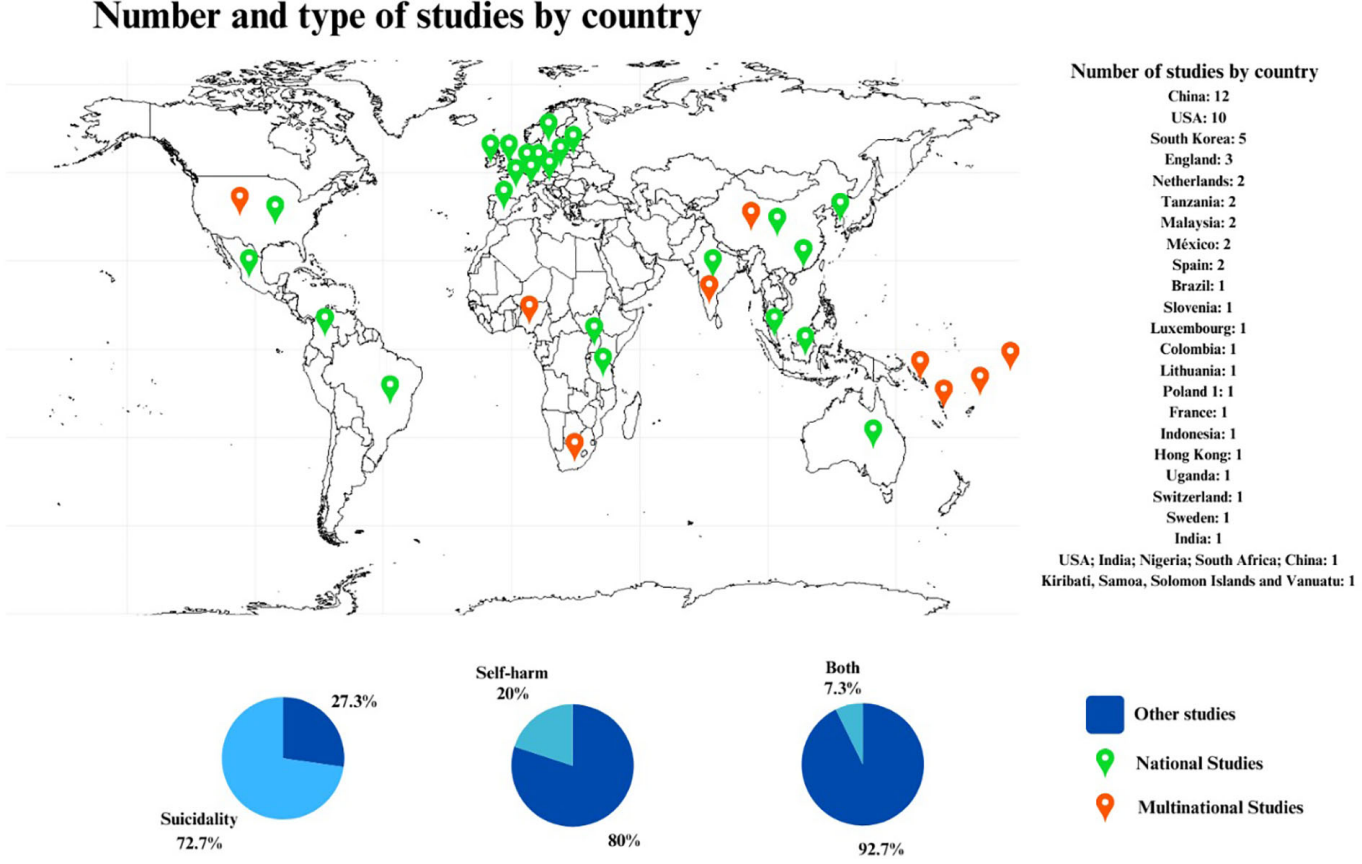
Map showing suicidality and self-harm study distribution by country, highlighting research focus on suicidality.

The 55 studies included a total of 2,109,801 participants. Among the 30 studies covering the pre-pandemic period, 12 did not report mean ages (40% of the sample). In the 25 studies from the after-pandemic group, 7 did not provide this information (28% of the sample). The mean age was 15.08 years with a standard deviation of 1.23 for the articles allocated to the pre-pandemic group, and a mean age of 15.08 years with a standard deviation of 1.47 for the post-pandemic group, resulting in an overall mean age of 15.08 years with a standard deviation of 1.34. The age range was 5-22 years. The predominant gender was female. The geographical distribution of the studies is illustrated in **Figure 2**, and the characteristics of the pre- and post-pandemic studies are detailed in **Tables 2**, **3**.

**Table 2:** Descriptive characteristics of included studies (Pre-Pandemic).

Reference, country, type of study	N / Age / Gender / Purposes	Prevalences of self-harm (NSSI) and/or suicidality	Main prevalence associations	Quality score
Alves Junior et al. (19) Brazil Cross-sectional	N = 1,118 Age: 14 – 19 Mean age: 16.0 Male: 613 (54.2%) Female: 519 (45.8%) Assess the prevalence of suicidal ideation, planning, and attempts, while identifying the sociodemographic and lifestyle factors among adolescents in Brazil's southern region.	Suicidal Thinking: 153 (13.8%) Suicidal Planning: 117 (10.5%) Suicidal Attempt: 62 (5.5%)	Adolescents who engage in suicidal behaviors frequently struggle with inadequate sleep and an unhealthy perception of their body weight.	100%
Bamwine et al. (20) USA Cross-sectional	N = 1,609 Age: 14 – 19 Mean age: 16.6 Male: 786 (49.1%) Female: 823 (50.9%) Investigate the prevalence of homicide survivals and their connections to suicidality and childhood adversities in a representative group of adolescents.	Suicidal Ideation: 16% among homicide survivors vs. 9% among non-survivors. Suicide Attempts: 11% (N = 179) among homicide survivors vs. 4% (N = 64) among non-survivors	After adjusting for demographic factors, homicide survival was linked to suicide attempts, though this association lost significance when childhood adversities were also considered. This indicates that exposure to numerous childhood adversities may play a key role in raising the risk of suicide attempts among those who have survived homicides.	100%
Bracic et al. (21) Slovenia Cross-sectional	N = 1,547 Age: 15 Mean age: 14.9 Male = 708 (46.1%) Female = 839 (53.9%) Determine the occurrence and contributing factors of suicidal ideation among adolescents in Slovenia.	Suicidal thoughts: N = 240 (15.5%) M = 178 (21.2%) F = 62 (8.8%)	Depressive symptoms, increased loneliness, frequent health issues, lack of family support, and involvement in bullying; either as victims or perpetrators; are all linked to suicidal ideation in adolescents.	100%
Cheng et al. (22) Baltimore, MD; New Delhi, India; Ibadan, Nigeria; Johannesburg, South Africa; Shanghai, China Cross-sectional	N = 2,393 Age: 15 - 19 Mean age: 16.76 Male = 1,227 (51.2%) Female = 1,112 (48.8%) Examining the connection between social support and the mental well-being of at-risk adolescents.	The prevalence of suicidal ideation varied between 7.9% and 39.6% across the countries, while suicide attempts ranged from 1.8% to 18.3%. Suicide attempts: (Male+Female = N) Baltimore 119 + 174 = 293 New Delhi 100 + 43 = 143 Ibadan 437 + 342 = 779 Johannesburg 282 + 239 = 521 Shanghai 67 + 141 = 208	Inadequate family support and weak ties to the community contribute to poor mental health outcomes, including suicidal ideation.	100%
Heinz et al. (23) Luxembourg Cross-sectional	N = 5,262 Age: 12 - 18 Mean age: not mentioned Male = 2,481 (47.3%) Female = 2,764 (52.7%) This research aims to determine whether the Health Behaviour in School-aged Children Symptom Checklist (HBSC-SCL), which assesses eight subjective health complaints like headaches and low mood, can effectively serve as a screening tool for detecting suicidal thoughts and behaviors in adolescents.	Thought about suicide in the past 12 months: 778 (15.1%) Planned suicide in the past 12 months: 722 (14.1%) Attempted suicide in the past 12 months: 392 (7.6%)	The eight components of the HBSC-SCL, including headache, stomach-ache, back pain, feeling low, irritability, nervousness, sleep disturbances, and dizziness, are linked to suicidality. Each of these items shows a statistically significant correlation with the four SIB (Suicidal and Ideation Behavior) items, although the strength of these correlations varies.	100%
Ibrahim et al. (24) Malaysia Cross-sectional	N = 1,769 Age: 13 – 17 Mean age: 14.4 Male: 838 (47.4%) Female: 931 (52.6%) Determine the prevalence and key predictors of depression and suicidal ideation among secondary school students in Malaysia	Suicidal ideation: Male: 214 (25.6%) Female: 279 (30.0%)	Suicidal ideation is significantly more prevalent among bullying victims and adolescents showing depressive symptoms, with a rate of 39.3%	100%
Kim et al. (25) South Korea Cross-sectional	N = 191,642 Age: 12 – 18 Mean age: not mentioned Male: 95,800 (49.9%) Female: 95,842 (50.1%) Investigate the hypothesis that Korean adolescents exhibiting one of three specific sleep patterns are more likely to experience suicidality compared to those without such sleep disturbances. Data were drawn from the 2011 – 2013 Korea Youth Risk Behavior Web-based Survey.	Suicidal ideation: M = 12,955 (13.6%) F = 21,194 (22.1%) Suicidal plan: M = 4,400 (4.6%) F = 6,804 (7.1%) Suicide attempt: M = 2,408 (2.5%) F = 4,904 (5.1%)	Sleep problems were generally linked to suicide-related behaviors, with both awakening and bedtime patterns showing a U-shaped relationship, like findings in Korean adults.	100%
Lee et al. (26) South Korea Cross-sectional	N = 73,238 Age: 12 - 18 Mean age: not mentioned Male: 38,391 (52.41%) Female: 34,847 (47.59%) Examine the variations in suicidal behaviors based on parental marital status.	Suicidal ideation: N = 14,011 M = 5,855 (15.3%) F = 8,156 (23.4%) Suicide attempt: N = 3,616 M = 1,391 (3.6%) F = 2,225 (6.4%)	Parental remarriage seems to pose a notable risk for suicidal behaviors in adolescents, with a more pronounced effect on girls. Additionally, low family wealth is linked to an increased likelihood of suicidal thoughts and attempts among girls.	100%
Liu et al. (27) China Cross-sectional	N = 5,696 Age: 12 – 18 Mean age: 15.0 Female: 100% Investigate whether the onset of menarche and menstrual issues are linked to non-suicidal self-injury among female adolescents.	The lifetime prevalence and prevalence in the last year of NSSI were 28.1% (N = 1600) and 21.4%, respectively	A link between menarche and NSSI is driven by the rapid physical and hormonal changes, along with the psychosocial stress that accompanies the start of menstruation, making adolescents more susceptible to emotional dysregulation and raising the risk of NSSI.	100%
Liu et al. (28) China Longitudinal	N = 11,831 Age: 12 – 18 Mean age: 15.0 Male = 6,018 (51%) Female = 5,813 (49%) Investigate the prevalence, features, and risk factors of suicidal behaviors, encompassing ideation, planning, and attempts.	Total of suicide attempts N = 309 M = 127 (41.1%) F = 182 (58.8%)	Being female, along with factors such as smoking, alcohol use, internalizing and externalizing issues, feelings of hopelessness, a history of suicide among friends or acquaintances, low family income, and poor relationships with parents, were all strongly linked to a higher risk of suicidal behavior.	100%
Mitchell et al. (29) USA Cross-sectional	N = 1,560 Age: 10 - 17 Mean age: 14.5 Male: 775 (49.6%) Female: 785 (50.4%) Investigate the relationship between exposure to websites promoting self-harm or suicide and the presence of self-harm or suicidal thoughts.	NSSI: 5% (N = 77) had thoughts of self-harm in the past month Suicidality: 2.5% (N = 39) had thoughts of suicide in the past month	Accessing websites that promote self-harm or suicide is linked to an increased likelihood of having self-harm or suicidal thoughts.	100%
Morey et al. (30) England Cross-sectional	N = 2,000 Age: 13 - 18 Mean age: 13.0 Male = 957 (47.85%) Female = 1,043 (52.15%) Investigate the relationship between self-harm and overall well-being.	Total of participants who ever self-harmed: 309 (15.5%) F = 241 (23.1%) M = 68 (7.1%)	Self-harm has a higher prevalence in girls. The link between self-harm and mental well-being indicates a strong connection to emotional distress. Girls who engage in cutting on body areas beyond the arms should be prioritized to intervention, as these actions are tied to higher emotional risk factors.	100%
Orozco et al. (31) Mexico Cross-sectional	N = 28,519 Age: 12 - 17 Mean age: not mentioned Male: 14,231 (49.9%) Female: 14,288 (50.1%) Outline the national prevalence of suicide attempts among Mexican students and assess the strength of the relationship between suicide attempts and four academic performance indicators, while controlling for other sociodemographic factors.	Lifetime suicide attempts: 3.0% for high school students, 4.2% for elementary school students. Suicide attempts were more prevalent among girls (5.2%) in high school and 6.8% in elementary school than among boys, 1.1% in high school and 1.5% in elementary school. Number of suicide attempts: 1,054	Academic performance factors, including poor self-perceived academic performance and a higher incidence of failures, were linked to suicide attempts. Students with poorer academic performance were more likely to attempt suicide, indicating a potential dose-response relationship across educational levels. Not being enrolled in the previous year and failing three or more school years were strong predictors of suicide attempts in high school students.	100%
Park and Lee (32) South Korea Cross-sectional	N = 727 Age: 12 – 18 Mean age: 15.06 Male: 376 (51.72%) Female: 351 (48.28%) Investigate the underlying reasons for suicide attempts among individuals from multicultural families in Korea.	Total number of suicide attempts: N = 41 (5.6%)	Foreign-born parents, being distant from family, and experiencing conflicts with a teacher contribute to a higher risk of suicide	100%
Peltzer and Pengpid (33) Kiribati, Samoa, Solomon Islands and Vanuatu Cross-sectional	N = 6,540 Age: 13 – 16 Mean age: not mentioned Male: 3,355 (51.3%) Female: 3,185 (48.7%) Examine the connection between early substance use, including alcohol, cannabis, and smoking, and its association with suicidal ideation and attempts.	Suicidal ideation: N = 25.8% Male = 26.8% Female = 24.4% Suicide attempt: N = 2,282 (34.9%) Male = 36.7% Female = 31.9%	Starting smoking, alcohol consumption, and drug use at an early age are linked to an increased risk of suicidal behaviors.	100%
Prairie et al. (34) USA Cross-sectional	N = 83,852 Age: 15 - 18 Mean age: 16.0 (SD = 1.2) Male: 41,506 (49.5%) Female: 42,346 (50.5%) Analyze the impact of including sexual orientation as a protected category in state hate crime laws on the reduction of suicide attempts among high school students.	Suicide attempts: N = 5,282 from 2015 - 2018	Hate crime laws including sexual orientation have been linked to a significant 1.2 percentage point decrease in suicide attempts among adolescents. The reduction was more pronounced among questioning and bisexual youths compared to their gay and lesbian peers.	100%
Roche et al. (35) USA Cross-sectional	Study 1: N = 547 Study 2: N = 340 Study 1: 11 – 15 Mean age: 12.8 Study 2: 15 – 18 Mean age: 16.37 Study 1: Male: 247 (45%) Female: 300 (55%) Study 2: Male: 177 (51.8%) Female 163 (48.2%) Investigate how differences in family immigration status influence Latino adolescents' reactions to immigration policies and news, and how these responses impact their overall adjustment.	Study 1: 14.5% (N = 77) reported suicidal ideation in the last 6 months.	Adolescents from families with more precarious immigration statuses exhibited heightened psychological and behavioral response to immigration issues. These challenges, including withdrawal behaviors, were linked to increased internalizing and externalizing symptoms, higher rates of substance use, and elevated suicidal ideation.	100%
Ruiz-Robledillo et al. (36) Spain Cross-sectional	N = 1,386 Age: 11 - 19 Mean age: 13.42 Male = 698 (50.4%) Female = 688 (49.6%) To describe suicidal behavior and assess the connection between family and school environments and suicidal behavior, considering the potential mediating roles of depression and anxiety.	Suicidal ideation: N = 114 (8.2%) F= 61 (8.9%) M= 53 (7.6%) Suicide plan: N = 72 (5.2%) F = 39 (5.7%) M = 33 (4.7%) History of suicide attempt: N = 51 (3.7%) F = 30 (4.4%) M = 21 (3%) Suicide attempt: N = 71 (5.1%) F = 43 (6.3%) M = 28 (4%) More than one attempt: N = 73 (5.3%) F = 40 (5.8%) M = 33 (4.7%)	Depression, anxiety, and suicidal behavior are interconnected. Poor family communication is associated with a negative school climate and is positively linked to depression, anxiety, and suicidal behavior. Conversely, a positive school climate is inversely related to these mental health challenges.	100%
Shayo and Lawala (37) Tanzania Cross-sectional	N = 3,793 Age: 13 – 17 Mean age: not mentioned Male: 1,819 (47.9%) Female: 1,974 (52.1%) Assess the likelihood of suicidal behavior being connected to experiences of school bullying.	Total suicidal ideation: N = 536 (14.1%) Total suicide attempts: N = 422 (11.1%) Ages 13 – 17 Total suicidal ideation: N = 380 (69.8%) Total suicide attempts: N = 311 (72.3%) Suicide attempts: N = 71 (5.1%) F = 43 (6.3%) M = 28 (4%) More than one attempt: N = 73 (5.3%) F = 40 (5.8%) M = 33 (4.7%)	Being a victim of bullying was identified as a predictor of suicidal ideation and attempts among adolescents in school.	100%
Shayo and Lawala (38) Tanzania Cross-sectional	N = 3,793 N 13 - 17y = 2,987 Mean age: not mentioned N 3,793: Male = 1,819 (47.9 %) Female = 1,974 (52.1%) N 13 - 17y 2987: Male = Not mentioned Female = Not mentioned Assess the relationship between food insecurity and suicidal behaviors among school-aged adolescents, considering the influence of common psychosocial factors.	Suicidal ideation: 14.1%; Suicide attempt: 11.1% N 13 - 17y 2987 Suicide ideation N = 380 (12.72%) Suicide attempt N = 311 (10.41%)	Adolescents facing food insecurity showed a significantly higher likelihood of experiencing suicidal thoughts and attempts. The stress, anxiety, and shame associated with food insecurity can aggravate mental health issues, elevating the risk of suicidal behaviors.	100%
Stallard et al. (39) England Longitudinal	N = 3,964 Age: 12 - 16 Mean age: not mentioned There were losses across the time, not allowing to measure the number of males and females’ participants Investigate the prevalence of self-harm in early adolescents and the factors that influence the development and continuation of this behavior over a one-year period.	Total number of self-harm thoughts N = 27% at some point in the past year Self-harm N = 594 (15%) at some point in the past year.	Frequent exposure to bullying doubled the risk of developing self-harm thoughts, while cannabis use heightened the likelihood of persisting with these thoughts. Conversely, a stronger sense of school belonging was associated with reduced risk of self-harming thoughts.	100%
Suárez Colorado and Campo-Arias (40) Colombia Cross-sectional	N = 339 Age: 13 – 19 Mean age: 16.3 Male = 143 (42.2%) Female = 196 (57.8%) Investigate the relationship between trust, communication, and feelings of alienation with suicide risk among school-aged adolescents in Colombia.	30% prevalence of suicide risk	A link was found between elevated suicide rates and low levels of trust or poor communication with either the mother or father.	100%
Tang et al. (41) China Cross-sectional	N = 15,623 Age: 12 – 18 Mean age: 15.2 Male = 8,043 (51.1%) Female = 7,580 (48.5%) Carry out a nationwide survey to investigate the prevalence and identify the risk factors for non-suicidal self-injury among adolescents in China.	Non-suicidal self-injury (5 times or more): N = 1,908 M = 951 (49.8%) F = 957 (50.2%) Pre-self-injury (1 to 4 times): N = 2,651 M = 1,296 (48.9%) F = 1,355 (51.1%)	Female, minor ethnicity, being the only child in the family, father's education, neglect, maltreatment, loneliness, lack of social support, bad emotional management ability and suicidal behaviors were strongly linked to non-suicidal self-injury (NSSI).	100%
Thakur et al. (42) India Cross-sectional	N = 715 Age: 14 - 19 Mean age: not mentioned Male = 361 (51.2%) Female = 344 (48.8%) Assess the prevalence of suicidal thoughts and investigate the factors that predict their occurrence.	N = 218 had suicidal ideation (30.9%). Of these: F = 128 (37.2%) M = 90 (24.9%) 121 aged 16 – 19 years (37.8%) 97 aged 14 – 15 years (25.2%)	Adolescents facing family-related challenges, verbal or physical abuse, and concerns about body image showed higher odds of experiencing suicidal thoughts. The prevalence was higher among females, possibly due to feelings of embarrassment in discussing these thoughts, which led to a sense of social isolation.	100%
Toomey et al. (43) USA Cross-sectional	N – 120,617 Age: 11 – 19 Mean age: 14.7 Female: 60,973 (50.6%) Male: 57,871 (48%) Transgender, Male to Female: 202 (0.2%) Transgender, Female to Male: 175 (0.1%) Transgender, Not Exclusively Male or Female: 344 (0.3%) Questioning: 1,052 (0.9%) This study aimed to examine the prevalence of suicidal behavior across six gender identity groups: female, male, transgender female (MTF), transgender male (FTM), transgender non-binary, and questioning. Furthermore, it explored how differences in key sociodemographic factors influence suicidal behavior within these identity categories.	14.1% of adolescents (N = 17,007) in the sample reported that they had ever tried to kill themselves one or more times	Transgender populations have an increased risk to suicide.	100%
Van Vuuren et al. (44) Netherlands Cross-sectional	N = 8,499 Age: 13 - 16 Mean age: not mentioned Not mentioned the number of males and females’ participants Assessing the progression of suicidal thoughts and behaviors (STBs) among Dutch multi-ethnic students at two key ages: 13 – 14 years (t1) and 15 – 16 years (t2).	Suicidal thoughts at t1: 12.7%. Suicide attempts at t1: 1.7% (N = 144) Suicidal thoughts at t2 for those with suicidal thoughts at t1: 26.3%. Suicide attempts at t2 for those with suicide attempts at t1: 12.5% (N = 1,062)	Suicidal thoughts and behaviors (STBs) were common in adolescents, they typically fade within two years. However, a subset of adolescents, particularly those facing psychosocial challenges and living in an unsafe environment, continuous experience STBs, placing them at greater risk for suicide.	100%
Van Vuuren et al. (45) Netherlands Longitudinal	N = 26.273 Age: 13 - 14 Mean age: 13.6 Not mentioned the number of males and females’ participants Analyze the 5-year trends in suicidal thoughts and attempts among adolescents, while exploring potential variations in these trends across different sociodemographic groups.	Suicidal thoughts: 17.6% (2010 - 2011) to 13.2% (2014 - 2015) Suicide attempts: N = 630 (2.4%) 2.9% (2010 - 2011) to 1.9% (2014 - 2015)	Adolescents of Surinamese, Turkish, and Moroccan descent showed a notable decline in suicidal thoughts, while the rates for Dutch-origin students remained relatively stable. Similarly, the largest reductions in suicide attempts were observed among adolescents from Turkish and Moroccan backgrounds.	100%
Yun et al. (46) USA Cross-sectional	N = 265,268 Age: 13 - 18 Mean age: not mentioned Male: 133,987 (50.51%) Female: 129,398 (49.49%) Investigate racial and ethnic disparities in risk behaviors associated with injuries and deaths among adolescents in Missouri, with a particular emphasis on Hispanic adolescents. Missouri Youth Risk Behavior Survey (YRBS) was used.	Considered suicide: 23.0% (Hispanics) 14.2% (non-Hispanic whites) 11.4% (non-Hispanic blacks) Planned suicide: 20.0% (Hispanics) 12.2% (non-Hispanic whites) 7.4% (non-Hispanic blacks) Attempted suicide: 17.8% (Hispanics) 6.0% (non-Hispanic whites) 5.4% (non-Hispanic blacks) Considered suicide: N = 1,585 (14.2%) Planned suicide: N = 1,584 (12.1%) Attempted suicide: N = 1,458 (6.9%)	Hispanic adolescents exhibit a higher rate of injury-related risk behaviors, including feelings of sadness or hopelessness, suicidal ideation, planning, and attempts, compared to their non-Hispanic White and Black counterparts.	100%
Zaborskis et al. (47) Lithuania Cross-sectional	N = 3,572 Age: 13 - 15 Mean age: not mentioned Male = 1,805 (50.5%) Female = 1,767 (49.5%) Examine the prevalence of suicidal ideation and attempts, along with their associations with various family factors	Considered the attempt of suicide: N = 844 (23.8%) M = 297 (16.6%) F = 547 (31.1%) Planning: N = 486 (13.7%) M = 195 (10.9%) F = 291 (16.5%) Attempt: N = 471 (13.2%) M = 205 (11.4%) F = 266 (15.1%) Attempted suicide: N = 1458 (6.9%)	Adolescents in Lithuania are more likely to experience suicidal ideation and attempts when coming from non-intact families or those with poor family functioning, highlighting these as significant predictors.	100%
Zygo et al. (48) Poland Cross-sectional	N = 5,685 Age: 13 – 19 Mean age: 16.91 Male = 1,705 (30%) Female = 3,980 (70%) The objective was to assess the occurrence of suicidal thoughts, tendencies, and attempts in young people, while also identifying the key factors that these individuals believe contributed to their suicide attempts.	Suicidal ideation: N = 1269 24.66% F = 1026 (28.57%) M = 243 (15.63%) Suicidal plans: N = 797 (15.55%) F = 646 (18.04%) M = 151 (9.77%) Suicide attempts: N = 225 (4.37%) F = 192 (5.34%) M = 33 (2.14%)	Girls were more likely to attempt suicide due to feelings of helplessness, loneliness, rejection, guilt, and conflicts with parents or peers, while boys were more often driven by peer pressure or online interactions. Suicide attempts were notably higher among girls aged 13 – 19 and more frequent in urban settings. Adolescents who experienced suicidal thoughts, plans, or attempts were more often raised in single-parent households and were more likely to report parental alcohol abuse and experiences of psychological or physical violence within the family.	100%

**Table 3:** Descriptive characteristics of included studies (Post-Pandemic).

Reference, country, type of study	N / Age / Gender / Purposes	Prevalences of self-harm (NSSI) and/or suicidality	Main prevalence associations	Quality score
Bukuluki et al. (49) Uganda Cross-sectional	N = 219 Age: 13 - 19 Mean age: not mentioned Male: 71 (32%) Female:148 (68%) The goal was to evaluate suicidal ideation and attempts among adolescents considered to be at higher risk.	Suicide ideation in the past 4 weeks: N = 67 (30.6%) F = 50 (33.8%) M = 17 (23.9%) Suicide ideation in the past 1 week: N = 28 (13.3%) F = 23 (16.1%) M = 5 (7.4%) Suicide plan for suicide in the past 1 week: N = 21 (9.6%) F = 17 (11.5%) M = 4 (5.6) Ever attempted suicide: N = 101 (46.1%) F = 71 (48.0%) M = 30 (42.3%) Attempted suicide in the last 6 months: N = 53 (24.2%) F = 35 (23.6%) M = 18 (25.4%)	Financial hardship, living apart from biological family or in fractured family environments, and psychological distress are the primary drivers of the high prevalence of suicidal ideations and attempts.	100%
Cheng et al. (50) China Cross-sectional	N = 18,900 Age = 12 - 18 Mean age: 14.99 Male: 9,416 (49.8%) Female: 9,880 (52.3%) Investigate the direct impact of cell phone addiction on suicidal behavior, as well as the indirect influence of poor sleep quality.	Number of cases according to the stage of suicidality Ideation M = 778 (8.3%) F = 1,238 (13.1%) Planning M = 586 (6.2%) F = 997 (10.5%) Attempt M = 362 (5.2%) F = 644 (5.5%)	Cell phone addiction is directly linked to suicidal ideation and planning, while poor sleep quality, female gender, and being a rural adolescent are associated with an increased risk of suicidal behavior.	100%
Dumont et al. (51) Switzerland Cross-sectional	N = 492 Age: 14 - 17 Mean age: 15.4 Male: 234 (48%) Female: 258 (52%) The study explores the prevalence of suicidal ideation among adolescents during the COVID - 19 pandemic and highlights both direct and indirect risk factors linked to this issue.	Presented suicidal ideation: N = 71 (14.4%) F = 53 (75%) M = 18 (25%) Attempted suicide: 5 (1,0%)	Girls had a notably higher likelihood of experiencing suicidal ideation, as did adolescents identifying as lesbian, gay, or bisexual (LGB), or those facing high psychological distress, low self-esteem, limited social support, academic struggles, or bullying. Heavy social media use, smoking, alcohol consumption, or significant impact from the pandemic showed an increase in suicidal ideation.	100%
Geulayov et al. (6) England Cross-sectional	N = 10,460 Age: 12 – 18 Mean age: not mentioned Male = 3,856 (36.9%) Female = 6,604 (63.1%) The objective was to assess the prevalence of loneliness and self-harm in a large, diverse community sample of 10,460 adolescents aged 12 – 18, following the initial months of the COVID - 19 pandemic, and to explore the relationship between loneliness, changes in loneliness during lockdown, and self-harm. Loneliness and self-harm were self-reported by participants.	Lifetime self-harm: N = 1,452 (13.9 %) M = 283 (7.3%) F = 1,169 (17.7%) Past year self-harm: N = 1,129 (10.8%) M = 204 (5.3%) F = 925 (14.0%) Past six months: N = 879 (8.4%) M = 152 (3.9%) F = 727 (11.0%) Self-harm during 1st UK lockdown: N = 787 (7.5%) M = 135 (3.5%) F = 652 (9.9%)	Shifts in loneliness are linked to an increased risk of self-harm. Loneliness interventions might be a potential target for intervention.	100%
Hou et al. (52) China Cross-sectional	N = 761 Age: 14 - 18 Mean age: 16.09 Male = 451 (59.3%) Female = 310 (40.7%) The objective is to explore the prevalence of suicidal ideation and attempts while analyzing the similarities and differences in the influencing factors between left-behind children and their non-left-behind peers.	Suicidal ideation: N = 277 (36.4%) Suicide attempt: N = 79 (10.4%)	Girls exhibited a higher susceptibility to suicidal ideation compared to boys in both groups. Adolescents whose parents had higher education levels showed a greater likelihood of suicidal ideation. Those with average or below-average financial conditions had a lower risk of suicidal ideation. Maladaptive coping strategies were linked to an increased risk of suicidal ideation, while anxiety and depression were also associated with a potential heightened risk.	100%
Jollant et al. (53) France Longitudinal	N = 85,679 Age: 10 - 80+ Mean age: not mentioned Male = 31,955 (37.2%) Female = 53,724 (62.8%) Even though the article evaluates ranges, like 10 – 19 years, it doesn’t specify the number for this population, neither the number of males nor females Examine the lasting effects of the COVID - 19 pandemic on individuals admitted to hospitals for self-harm.	Number of hospitalizations for self-harm in France from September 2020 to August 2021 N = 20,964 M = 3,794 (18%) F = 17,170 (82%)	The number of self-harm hospitalizations declined overall. However, variations were observed based on age and gender.	93,75%
Lee and Hong (54) Korea Cross-sectional	N = 167,099 Age: 12 - 18 Mean age: 15.0 Male = 82,679 (51.9%) Female = 76,510 (48.1%) 2019 M = 28,084 (51.8%) F = 25,964 (48.2%) 2020 M =27,199 (52.0%) F = 25,295 (48.0%) 2021 M = 27,396 (51.9%) F = 5251 (48.1%) This study explored the short-term (2020) and long-term (2021) effects of the COVID - 19 pandemic on suicide-related behaviors among Korean adolescents, comparing them to the pre-pandemic year (2019), while also analyzing the factors contributing to these changes, based on data from the Korea Youth Risk Behavior Web-based Survey.	Suicidal ideation: N = 26,207 (12.2%) 2019 N = 6,871 (12.7%) 2020 N = 5,619 (10.7%) 2021 N = 6,535 (12.4%) Suicide planning: N = 8,116 (3.7%) 2019 N = 2,000 (3.6%) 2020 N = 1,794 (3.4%) 2021 N = 2,010 (3.8%) Suicide attempts: N = 5,208 (2.4%) 2019 N = 1,474 (2.6%) 2020 N = 1,007 (1.9%) 2021 N = 1,125 (2.1%)	The initial decline in mental health issues during 2020 could be attributed to reduced academic pressures and fewer interpersonal conflicts caused by school closures. However, the worsening mental health in 2021 emphasizes the need for ongoing interventions, particularly for vulnerable adolescents, such as those from lower socioeconomic backgrounds.	100%
Meeker et al. (55) USA Cross-sectional	N = 1,532 Age: 14 - 18 Mean age: not mentioned Male: 735 (48%) Female: 797 (52%) Analyze the prevalence of Adverse Childhood Experiences (ACEs) in a community sample of adolescents and investigate how exposure to multiple ACEs influences various health risk behaviors.	NSSI: Non-suicidal self-injury (NSSI) report: 18.9% of youths with two or more ACEs versus 3.5% of those without ACEs. Suicidality: Suicidal ideation: 30.3% of youth with two or more ACEs versus 4.7% of those without ACEs Suicide attempt: 15.4% of youth with two or more ACEs versus 0.9% of those without ACEs	Adolescents with multiple ACEs were significantly more likely to report mental health symptoms, suicidal thoughts, violent behavior, and substance use. The accumulation of traumatic experiences is linked to several health risk factors during adolescence.	100%
Mohd Fadhli et al. (8) Malaysia Cross-sectional	N = 1,290 Age: 13 - 17 Mean age: 14.48 Male = 385 (29.8%) Female = 905 (70.2%) Assess the prevalence of cyberbullying and suicidal behavior among adolescents in Peninsular Malaysia and examine the relationship between these two factors.	Suicidal behavior: N = 221 (17.1%) Suicidal thought: N = 154 (11.9%) Suicide plan: N = 132 (10.2%) Suicide attempt: N = 108 (8.4%)	Link between being a victim of cyberbullying and an increased chance of exhibiting suicidal behavior. Adolescents who experienced cyberbullying were more likely to show suicidal tendencies. Additional risk factors were younger age, female gender, presence of depression, a history of abuse, and witnessing parental conflicts.	100%
Nguyen et al. (56) USA Cross-sectional	N = 13,605 Age: 12 - 18 Mean age: 15.0 Male: 6,690 (50.64%) Female: 6,862 (49.37%) This study explores the relationship between in-school and electronic bullying and suicide-related behaviors, as well as feelings of despair among adolescents, accounting for sociodemographic factors, abuse history, risk-taking behaviors, and physical appearance/lifestyle. Data comes from the 2019 U.S. Youth Risk Behavior Surveillance System (YRBSS) national survey.	Considered suicide: N = 755 (18.5%) Planned suicide: N = 1,306 (15.7%) Attempted suicide at least once: N = 1,018 (8.9%) Suicidal tendencies: N = 7,912 (58.5%)	An association was found between bullying and depressive symptoms with both conditions being linked to an increased risk of suicidality.	100%
Park et al. (57) Korea Cross-sectional	N = 227,139 Age: 12 - 18 Mean age: not mentioned 2018-2019 Male before Covid-19 = 60,304 (52.0%) 2020-2021 Male during Covid-19 = 56,754 (51.8%) 2018-2019 Female before Covid-19 = 57,039 (48.0%) 2020-2021 Female during Covid-19 = 53,042 (48.2%) Investigate the impact of the COVID - 19 pandemic on the mental health of students in Korea.	Suicidal ideation: 2018-2019 M = 9.5% F = 17.3% 2020-2021 M = 8.8% F = 15.0% 2019 M = 9.4% F = 17.1% 2020 M: 8.1% F: 13.9%	Depressive symptoms, suicidal thoughts, and stress were more prevalent among adolescents who did not live with their families, had low socioeconomic status, or perceived their health as poor.	100%
Peng et al. (58) China Cross-sectional	N = 39,751 Mean age: 14.79 Male: 18,966 (47.7%) Female: 20,785 (52.3%) Examine the connection between homeschooling during the pandemic and the risks of anxiety, depression, and suicide among elementary and high school students.	15.8% of men and 24.4% of women reported having suicidal thoughts in the two weeks prior to the date the study questionnaire was administered. 1,006 (5.3%) were suicide attempters	Girls were particularly affected during the pandemic. Sleep quality and time spent online influenced mental health. Strategies aimed at enhancing sleep and limiting exposure to pandemic-related content could help alleviate these negative impacts.	100%
Rogers et al. (4) USA Longitudinal	N = 102 Age: 10 - 19 Mean age: 16.0 Male = 80 (78.4%) Female = 22 (21.6%) Examine the effects of COVID - 19 on adolescent suicide rates in Maryland from 2019 to 2021.	Number of deaths by suicide among adolescents: 2019: N = 37 cases 2020: N = 31 cases 2021 N = 34 cases	Economic hardships and social isolation were risk features. When evaluating by year, there was a reduction in the number of suicides. Closer family moments seem like a protective feature. From 2020 to 2021 there is an increase, but no difference in comparison to the pre-pandemic period.	68,75%
Shankar et al. (59) USA Cross-sectional	Total N = 8,127 Mean age: not mentioned Before the pandemic: N = 5,278 During the pandemic: N = 2,849 Age: 5 - 17 Male: 41.5% Female: 58.5% Determine the causes for emergency department (ED) visits and the frequency of such visits during the COVID - 19 pandemic.	NSSI: Study links self-harm and suicide attempts: Pre-pandemic: N = 1,077 (20.4%) During the pandemic: N = 772 (27.1%) Total: N = 1,849 (22.8%) Suicidality: Study links self-harm and suicide attempts: Pre-pandemic: N = 1,077 (20.4%) During the pandemic: N = 772 (27.1%) Total: N = 1,849 (22.8%)	Disruptive, impulse-control, and behavioral disorders have seen a notable rise, potentially exacerbated by limited access to outpatient care and stressors brought on by the pandemic.	100%
Suárez Soto et al. (60) Spain Cross-sectional	N = 163 Age: 14 - 17 Mean age: 15.81 Male: 52 (32.5%) Female: 106 (65.6%) Did not respond: 3 (1.2%) 1 person identified as another gender (not specified) (0.6%) Investigate sociodemographic factors (gender, age, sexual orientation), victimization experiences (physical, psychological, and verbal abuse, violence, cyberbullying), and resilience factors linked to suicidal behavior among Spanish adolescents since the onset of the pandemic.	Out of 163, 45 reported involvements in some form of suicidal behavior F = 16 M = 28 20.8% of the participants expressed some thoughts, 22.6% considered a method/form of self-destruction. 7.4% attempted suicide. Suicidal ideation: F = 24 M = 9 Suicidal planning: F = 24 M = 13 Suicidal attempt: F = 10 M = 2	Individuals who experience psychological abuse are five times more likely to exhibit suicidal behavior; no significant link exists between gender and suicidal behavior, but there is a connection between sexual orientation and such behavior, with higher rates observed in the heterosexual group.	100%
Valdez-Santiago et al. (61) Mexico Cross-sectional	July 2018 - June 2019: N = 17,925 August-November 2020: N = 4,812 Age: 10 - 19 Mean age: not mentioned Does not separate gender in the total population; only in those who had suicide attempts. In 2018 - 2019: N = 333 Male = 77 (23.12%) Female = 256 (76.88%) 2020: N = 101 Male = 13 (12.8%) Female= 88 (87.2%) Examine the prevalence of suicide attempts among Mexican adolescents in the 12 months preceding and following the lockdown.	Prevalence of suicide similar in both studies. Pre-pandemic: 1.8% During the first month of the pandemic: 2.1%. Prevalence decreased in men in 2020 (0.3%), compared to 2018 - 19 (1%) Increase in women (3.8% in 2020 versus 2.7% in 2018 – 19). Prevalence among 10 – 14 years was 1.4% in both studies Increased prevalence among 15 – 19 years (2.3% to 2.8%). Suicide attempts in 2020: 101	Women, adolescents from urban areas who lost a family member to COVID, those in families affected by job loss, or who did not participate in online schooling are at a higher risk of attempting suicide.	100%
Wang et al. (5) China Longitudinal	T1: N = 3,588 T2: N = 2,527 Age T1: 15 - 18 Age T2: 16 - 18 Mean age: 16.13 Male = 1,217 (48.2%) Female = 1,310 (51,8%) Explore the effects of COVID - 19 and assess psychological risk factors among adolescents exhibiting non-suicidal self-injury (NSSI).	Self-harm emerging rate: N = 261 (10.3%) Sustained self-harm: N = 686 (27.2%) Total number of NSSI: 947	Reduced self-control, elevated neuroticism, and heightened impulsivity are linked to non-suicidal self-injury.	90,9%
Wang et al. (62) China Longitudinal	N = 5,854 Age: 10 - 19 Mean age: 12.04 Male: 3,020 (51.6%) Female: 2,834 (48.4%) Assess the mediating roles of depressive symptoms, emotional competence, and COVID - 19-related post-traumatic stress symptoms in the connection between family functioning and non-suicidal self-injury, based on data collected in 2020.	Total of adolescents who presented NSSI N = 1,768 (30.2%) 165 were hospitalized Bite (16.93%) Cut (15.70%) Scratch (13.75%)	Pandemic exacerbated students' vulnerability to NSSI, particularly among those with personality traits such as high neuroticism and impulsivity. It was found that there is a relationship between family functioning and NSSI; and that the variables depression, emotional competence, and PTSS-COVID19 had a significant mediating effect on this relationship.	90,9%
Yosep et al. (63) Indonesia Cross-sectional	2019: N = 175 2020: N = 268 Age: 14 - 18 Mean age: 13.2 2019: Male: 79 (45.7%) Female: 96 (53.3%) 2020: Male: 102 (38.1%) Female: 166 (61.9%) Examine the connection between bullying and the risk of suicide in adolescents.	Total of adolescents at risk of suicide 2020: F = 50 (71.4%) M = 20 (28.6%) 2019: N = 64 (it does not divide into men and women)	Both perpetrators and victims of bullying may face an elevated risk of suicide attempts or threats.	100%
Yu et al. (64) China Cross-sectional	N = 1,248 Mean age: 16.8 Male = 417 (33.41%) Female = 831 (66.59%) The objective of this study was to evaluate the current suicide risk and determine its connection to various forms of trauma, using data from the Chinese Youth Health-Related Behavior Questionnaire.	NSSI: Self-mutilation: N = 151 (12.10%) Suicidality: Suicidal ideation: N = 179 (14.34%) Suicidal plan: N = 103 (8.25%) Suicidal attempt: N = 102 (8.7%)	There is a higher prevalence of suicide risk (SR) among adolescents compared to the global adolescent suicide rate from previous two years ago, and higher than the findings in Chongqing in 2019, particularly among junior and senior high school students.	100%
Zetterqvist et al. (65) Sweden Longitudinal	T1 2011: N = 3,060 T2 2014: N = 5,743 T3 2020 - 2021: N = 3,258 Mean age T3: 18.19 Age: 16 - 18 T1: Female: 1,537 (50.2%) Male: 1,509 (49.3%) T2: Female: 3,153 (54.9%) Male: 2,536 (44.2%) T3: Female: 1,787 (54.8%) Male: 1,445 (44.4%) Examine the rise in non-suicidal self-injury rates throughout the pandemic.	Total number and percentage of lifetime prevalence of NSSI in adolescents in three time points: T1: N = 525 (17.2%) F = 402 (26.4%) M = 120 (8%) T2: N = 1.015 (17.7%) F = 778 (24.7%) M = 222 (8.8%) Non-binary: 15 (28.3%) T3: N = 898 (27.6%) F = 649 (36.3%) M = 231 (16%) Non-binary: 18 (69.2%)	Levels of depression and anxiety were elevated in 2020 – 2021 compared to 2014	93,75%
Zhao et al. (66) China Longitudinal	First wave N = 2,090 Second wave N = 1,609 Age: 12 - 18 Mean age: 16.5 Male: 588 (36.5%) Female: 1,021 (63.5%) Explore the relationship between PTSD symptoms during the pandemic and NSSI in adolescents over time. Analyze the mediating role of sleep problems and depressive symptoms in the connection between PTSD and NSSI.	Total number of adolescents who reported at least one episode of NSSI: N = 513 (31.9%) M = 169 (32.9%) F = 344 (67.1%)	Early PTSD symptoms persisted, influencing and forecasting future issues with sleep and depression, which subsequently heightened the likelihood of self-harming behaviors in adolescents.	95%
Zhou et al. (9) China Cross-sectional	N = 8,361 Mean age: 14.62 Male: 4,397 (52.58%) Female: 3,964 (47.41%) Examine the relationship between non-suicidal self-injurious behaviors, gender, coping strategies, and their interactions among Chinese students during the COVID - 19 pandemic.	Total number of adolescents who experienced NSSI: N = 476 (5.7%)	Coping strategies such as seeking social support, tolerance, emotional expression, and denial/fantasy are independent predictors of non-suicidal self-injury. Parental separation and enrollment in vocational high schools are associated with an increased likelihood of non-suicidal self-injury.	100%
Zhu et al. (7) China Cross-sectional	N = 5,175 Age: 9 - 19 Mean age: 13.38 Male = 2,673 (51.7%) Female = 2,502 (48.3%) An online cross-sectional survey was conducted to assess the prevalence of suicidal ideation (SI) during the COVID - 19 lockdown among Chinese adolescents and to examine gender-specific factors associated with SI.	The prevalence of suicidal ideation during the COVID - 19 pandemic lockdown of all the participants was 3%: F = 3.64% M = 2.39% Total number of adolescents with a history of previous suicide attempts: N = 610 (11.79%)	The prevalence of suicidal ideation (3%) among adolescents was lower compared to other studies (12.7%). Adolescent suicide rates did not increase during the COVID - 19 pandemic. Several factors may be related to home isolation may have reduced negative peer interactions; strain relationships with parents but returning to school may have minimized these conflicts.	100%
Zhu et al. (67) China, Hong Kong Longitudinal	N = 1,491 Age: 10 - 17 Mean age: 13.04 Male: 695 (46.6%) Female: 792 (53.4%) Examine shifts in suicidal episodes and explore the factors linked to suicidal ideation and recurrence among children and adolescents in Hong Kong.	Number of suicidal episodes Pre-pandemic (2019): 24% Experienced suicidal episodes only before the pandemic Total: 193 (14%) F = 85 (6.2%) M = 108 (7.8%) During the pandemic (2020): 21% Experienced suicidal episodes only during the pandemic Total: 148 (10.7%) F = 84 (6.1%) M = 64 (4.6%) Experienced episodes before and during the pandemic: Total: 143 (10.4%) F = 99 (7.2%) M = 44 (3.2%)	Depression, anxiety, stress, loneliness, social anxiety, a fixed mindset, reduced sense of life meaning, diminished self-control, and lack of parental support and supervision were key factors. Poor psychological well-being, limited family support, and the negative effects of the pandemic were consistently linked to suicidal ideation among students during this time.	100%

### 3.2 Prevalence profile of suicidality and non-suicidal self-injury over the past 14 years

Despite the shorter and more variable durations of post-pandemic study periods, a significant shift in self-harm prevalence was observed, particularly in NSSI. During the pre-pandemic period, spanning nine years (2010-2019 based on study time frames), data revealed that 45,233 adolescents attempted suicide, 5,025/yearly, representing 5.14% of the investigated population, while 4,411 adolescents engaged in NSSI, 490/yearly, accounting for 0.5%.

In contrast, the post-pandemic period (2020-2024 spanning 4 years), characterized by shorter and more heterogeneous study intervals, included datasets with varying temporal coverage. Aggregated data for the post-pandemic period revealed a significant reduction in suicide attempts, with 9,436 adolescents attempting suicide (0.76% of the population), 2,359/yearly. Conversely, NSSI prevalence surged, involving 29,970 adolescents, 7,492/yearly or 2.43% of the population.

To account for differences in study durations, the average annual prevalences were calculated. For suicide attempts, the pre-pandemic average was 0.57%, decreasing to 0.19% post-pandemic, representing a 66.67% reduction. Conversely, NSSI showed a significant increase, with the pre-pandemic average of 0.056% rising to 0.61% post-pandemic, corresponding to a 992.86% increase. These findings highlight a pronounced escalation in NSSI behaviors among adolescents during the post-pandemic period, despite the marked reduction in suicide attempts. Although the focus on suicidality has dominated the scientific agenda, recent findings underscore a significant and genuine rise in self-injury rates post-pandemic, reflecting the amplification of risk factors exacerbated by the pandemic context.

#### 3.2.1 Risk and protective factors across contexts

The main factors associated with self-harm and suicidality were identified through thematic frequency across the 55 included studies. Lack of family and social support was reported in 30 studies, physical and psychological health problems in 21, bullying involvement in 9, psychoactive substance use in 8, self-image/self-esteem issues in 5, and academic performance difficulties in 4. These counts indicate how frequently each factor was examined and reported across studies; no pooling of participant-level prevalence or effect estimates was performed due to heterogeneity of designs and measures.

### 3.3 Across time: changes in prevalences between the pre- and post-pandemic periods

During the period from 2010 to 2019, most studies (n=24, corresponding to 82% of the articles) reported a high prevalence of suicidality and non-suicidal self-injury (NSSI) among adolescents. Physical, psychological, contextual, and environmental factors were consistently associated with these behaviors. Notably, the lack of family support, bullying, mental health problems, and academic difficulties were key factors.

Gender also played a significant role, with a higher prevalence of suicidality among female adolescents, often linked to low self-esteem and family conflicts.

Regarding the 2020-2024 period, most reviewed studies (n=18, corresponding to 69.23% of the articles evaluated in this review) identified an increase in the prevalence of suicidality and self-harm among adolescents. Contributing factors included a lack of family support, psychological and physical problems, socioeconomic and environmental issues, as well as excessive use of psychoactive substances.

The COVID - 19 pandemic transformed the landscape of risk factors associated with suicidality and NSSI among adolescents, revealing new dimensions of mental vulnerability. Social isolation and increased loneliness, driven by prolonged school closures and social distancing measures, emerged as critical risk factors. Increased loneliness correlated strongly with self-harm risk, emphasizing the urgency of preventive interventions. Furthermore, intensified use of technology and social media, while serving as an outlet for many, exacerbated deteriorations in sleep quality, a factor directly linked to heightened suicidal ideation and self-harm behaviors.

This scenario was aggravated by financial instability and complex family dynamics. Families facing economic hardship and pandemic-related losses presented adolescents with an elevated risk of suicide and self-harm. Adolescents experiencing heightened levels of depression and anxiety during the pandemic showed intensified vulnerabilities, amplified by low self-esteem and lack of family support.

Gender remained a critical determinant, with a higher prevalence of suicidality and self-harm among female adolescents. Sexual orientation and gender identity also significantly influenced these behaviors, with higher rates of suicidal ideation among homosexual and transgender adolescents compared to their heterosexual and cisgender peers.

Finally, adolescents who face academic difficulties, bullying, or low self-esteem exhibited a substantial increase in suicidal ideation and self-harm behaviors.

## 4 Discussion

Risk factors for suicidality and non-suicidal self-injury (NSSI) among adolescents underwent significant changes during the COVID - 19 pandemic. Pandemic stressors, such as loneliness during social isolation and economic hardship, were strongly heightened the risk non-suicidal self-injury (NSSI) globally, as observed in countries like China, the United Kingdom, Uganda, and South Korea (6, 49, 50, 54). Contrary to widespread concerns about a potential surge in suicide-related behaviors during the COVID - 19 pandemic, evidence from numerous studies suggests a more stable pattern in adolescent suicidality. For instance, research conducted in Maryland revealed that adolescent suicide rates remained relatively consistent throughout the pandemic, with only minor fluctuations during specific periods (4). Similarly, a study in Mexico found no significant differences in the prevalence of suicide attempts between pre-pandemic and pandemic periods, further emphasizing the stability of these behaviors (61). In Korea, longitudinal analysis showed improvements in suicidal ideation and attempts during both short- and long-term pandemic phases, highlighting potential resilience among adolescents (54). However, the broader picture demands caution before assuming a homogeneous trend.

While this systematic review identified a significant rise in non-suicidal self-injury, particularly among female adolescents, it is essential to acknowledge that this trend was not universally accompanied by a parallel increase in suicide attempts or ideation. Several studies, including those conducted in the United States (4), Mexico (61), and South Korea (54), reported stability or even reductions in certain suicide-related outcomes. Such variability suggests that the impact of the pandemic on adolescent suicidality may have been modulated by a combination of regional policy responses, cultural attitudes toward mental health, differences in access to healthcare services, stigma surrounding self-reporting, and the specific temporal phase in which data were collected.

Well-known pre-existing stressors exacerbate the impact of new stressors, such as remote schooling, social isolation, and economic instability. These factors heightened loneliness, disrupted sleep patterns, and intensified anxiety and depression, particularly in adolescents already at risk (40, 50). The interplay between contextual and individual risk factors created heightened challenges, emphasizing the need for targeted interventions (51).

### 4.1 Environmental or contextual risk factors

#### 4.1.1 Family and socioeconomic challenges

Factors such as lack of family support, financial instability, and family disconnection, previously were linked to increased suicidality, became even more pronounced as economic hardships deepened and social isolation restricted access to coping resources (6, 40, 49, 50). These challenges created new layers of vulnerability, highlighting the effects of pre-existing stressors (51). Supportive family environments have been shown to reduce self-injury rates by fostering psychological resilience and mitigating depressive symptoms (68)​. Conversely, dysfunctions within the family, such as conflict, poor communication, and lack of parental supervision, exacerbate maladaptive behaviors and significantly contribute to psychological distress and suicidal behaviors among adolescents (67, 69).

The loss of primary caregivers due to COVID - 19-related deaths placed many adolescents in precarious living arrangements, often with unfamiliar or distant relatives. These new environments sometimes led to exploitation or continued cycles of abuse, exacerbating adolescents’ sense of grief and emotional instability (70–73). Parental stress fosters environments conducive to abuse. Economic hardships, job losses, and fears of COVID - 19 infection contributed to elevated levels of parental anxiety, which often manifested as aggressive or neglectful behaviors toward adolescents (71, 72). Parents struggling with substance abuse during lockdowns further diminished their caregiving capacities, creating an environment where physical and emotional maltreatment became more frequent and severe (74). This is consistent with earlier findings that households with pre-existing dysfunctions, including parental alcoholism, saw a marked increase in violence and neglect during prolonged periods of stress (75, 76).

Economic instability, including parental job loss and food insecurity, significantly heightens these risks, as adolescents facing household financial difficulties display higher rates of depressive symptoms, suicidal ideation, and suicide attempts (77–80). The experience of reduced access to healthcare compounds their mental health challenges (81, 82).

Cultural and ethnic dynamics introduced additional layers of complexity. Adolescents from ethnic minority groups and immigrant families faced increased vulnerability to suicidality (26, 35, 44, 81). Unemployment and financial uncertainty have been shown to increase the prevalence of suicidal behaviors, particularly among adolescents from low-income households (77, 83)​.

The interplay between chronic stressors and acute stressors such as pandemic-related disruptions add on the effects on depression and suicide-related behaviors (67, 84).

#### 4.1.2 Bullying

The COVID - 19 pandemic significantly altered the prevalence and dynamics of bullying behaviors. Traditional bullying generally decreased during lockdowns due to reduced peer interaction in physical school settings, a trend attributed to school closures and enhanced parental supervision at home (85, 86). However, this decrease was counterbalanced by a substantial increase in digital bullying, as adolescents turned to online platforms for social interaction during periods of isolation (85, 87). Cyberbullying often intensified in severity, with prolonged digital exposure augmenting risks of mental health deterioration, particularly in individuals with pre-existing conditions, such as anxiety and depression (8, 88). Digital bullying impact is profound and multifaceted, coursing with heightened anxiety, depression, and social withdrawal, conditions exacerbated by the anonymity and permanence of online harassment (87, 88). Victims of any bullying exhibited markedly higher rates of suicidal ideation and attempts, often accompanied by depressive symptoms and diminished self-esteem (21, 24, 56, 88). During and after the pandemic, both traditional and cyberbullying emerged as significant predictors of suicidal behaviors (8, 60). Again, specific groups, including low-income households, LGBTQ+ and racialized youth, faced disproportionate rates of cyberbullying, reflecting enduring patterns of identity-based victimization that were intensified during the pandemic (82, 87, 89).

Socioeconomic and cultural contexts played a crucial role in shaping bullying dynamics. In countries with stricter public health measures, traditional bullying rates declined due to increased teacher supervision and minimized unstructured peer interactions (85, 86). Cyberbullying trends varied significantly, with sharp increases reported in regions with extended lockdowns and less stringent anti-cyberbullying policies (87, 89).

### 4.2 Individual factors

#### 4.2.1 Psychiatric and psychological problems

Pre-existing mental health conditions such as anxiety and depression experienced significant worsening of symptoms due to the stress of the pandemic (90). Poor sleep quality and excessive screen time disrupted emotional regulation (50). Adolescents with difficulties regulating emotions often turned to maladaptive coping mechanisms like NSSI to manage overwhelming feelings of loneliness and helplessness during prolonged lockdowns (65). This pattern underscores the pivotal role of emotional regulation skills in mitigating the adverse effects of pandemic-related stress on mental health (73, 76).

Prolonged confinement in households where abuse was already present increased exposure to physical, emotional, and even sexual violence, as adolescents were isolated from potential protective buffers (71, 72). Adverse childhood experiences (ACEs) heightened emotional distress and reduced the ability of adolescents to adapt to changing circumstances (49, 55, 91). Accumulative exposure to ACE played a significant role in increasing susceptibility to self-harm and suicidal ideation. Low resilience emerged as a critical factor, who lacked robust coping mechanisms facing greater challenges in managing stress effectively (92). The ones with limited coping skills and emotional resources were less equipped to handle pandemic-related stressors, leaving them particularly vulnerable to suicidality and NSSI (92, 93). Difficulties in access to mental health resources, disproportionately affected underserved communities, intensifying their struggles with mental health (75, 76). Increases in self-injury behaviors were driven by a lack of effective coping strategies and emotional resilience (70).

#### 4.2.2 Substance use and media exposure

The use of psychoactive substances, including alcohol and tobacco, was frequently associated with suicidal behaviors, particularly in socially vulnerable adolescents (35, 51). Dependency on digital platforms also emerged as a significant factor, with adolescents experiencing higher rates of anxiety and depression due to constant comparison and exposure to harmful content online (50). Substance use and digital dependency appear to act as mechanisms for coping with stress and emotional distress, though they simultaneously exacerbate mental health vulnerabilities (94, 95).

When it comes to digital dependency, a phenomenon must be cited: Snapchat dysmorphia. It represents a growing phenomenon intricately linked to the interaction between social media usage, body self-image, and self-esteem, particularly among adolescents. Platforms like Snapchat, with their AI-driven filters, create unrealistic standards of beauty, often leading to distorted self-perceptions and behaviors aimed at achieving unattainable physical ideals (96, 97)​. Adolescents became particularly susceptible to body dissatisfaction and disordered eating behaviors, such as meal skipping and compulsive exercise, as they attempted to conform to the idealized images portrayed on these platforms (97)​. The appearance-based activities, including editing selfies and comparing oneself to filtered images, strongly correlate with depressive symptoms and body dissatisfaction, particularly in female adolescents (96)​. These activities perpetuate a cycle of negative self-evaluation, reinforcing the need for external validation and increasing vulnerabilities to mental health challenges (96). In the long-term, it seems to compromise identity formation, as constant exposure to idealized imagery contributes to maladaptive self-modeling and body image distortions (98)​. In extreme cases, adolescents use Snapchat as a platform to exchange extreme dieting goals and set unrealistic body standards. When these goals are not met, some engage in self-punishing behaviors, including self-harm, reflecting the pressures of peer influence and digital beauty culture (97, 98)​.

In a Saskatchewan study, the risk associated with co-occurring substance use was identified, particularly the simultaneous use of cannabis and alcohol. Adolescents with problematic use of both substances demonstrated a significant increase in suicidal ideation compared to those using only one substance (99). Recent changes in substance use patterns, such as increased frequency or quantity, were linked to heightened vulnerability, particularly among younger ones (99). Online gaming environments have been increasingly linked to heightened risks of problematic behaviors, including the consumption of alcohol. Adolescents, isolated due to pandemic restrictions, often turned to online gaming and alcohol consumption as maladaptive coping mechanisms to alleviate stress and loneliness (94, 95, 100). The reward systems in online gaming, coupled with peer interactions and challenges normalize or even incentivize risky behaviors like alcohol consumption (94, 95)​.

The interplay between gaming and substance use has been explained through the shared mechanism of escapism and coping strategies. Gamers, particularly those engaged in multiplayer games, are exposed to social interactions where alcohol consumption may be encouraged or rewarded, implicitly or explicitly (94, 95, 100)​. Anonymity and detachment provided by digital platforms, reduces the perceived consequences of risky behaviors (94, 95)​. The competitive and immersive nature of online gaming often leads to long sessions, in which players may resort to substances like alcohol to sustain engagement or bond socially, amplifying the risks of dependency and health issues (100).

These patterns are especially concerning for adolescents who are simultaneously navigating identity development and social relationships. Vulnerable groups, such as those already facing bullying or other social pressures, may find online gaming a double-edged sword, a refuge that inadvertently exposes them to additional risks, such as substance abuse (100).

#### 4.2.3 Self-image and self-esteem

Negative self-image and low self-esteem were closely linked to suicidality, particularly in adolescents facing body image challenges or social conflicts (19, 42). Personality traits such as neuroticism and impulsivity, which were amplified during the pandemic, further heightened vulnerability to self-injurious behaviors (5, 51).

Another interesting behavioral phenomenon is Fear of Missing Out (FoMO), which also plays a crucial role in the interaction between social media use, self-image, and self-esteem, particularly among adolescents. FoMO increases vulnerability to the negative influences of social media on self-image and self-esteem. The pervasive use of platforms amplifies feelings of inadequacy and exclusion, particularly when individuals perceive themselves as missing out on rewarding experiences shared by their peers (101). This phenomenon often drives excessive engagement with social media, contributing to a cycle of comparison and dissatisfaction with personal achievements or appearance (102). The adolescents from families with poor cohesion or rigid structures are more likely to seek validation and social gratification online, intensifying the risks associated with low self-esteem and distorted self-perception (101).

The impact of FoMO on adolescents’ mental health is worsened by societal pressures to conform to idealized standards of beauty and success frequently portrayed on social media. This exposure often exacerbates existing insecurities, particularly when with limited emotional regulation skills (103). As adolescents, they struggle to reconcile their perceived inadequacies with the curated realities of their peers (101).

#### 4.2.4 School-related stressors and risk of self-harm

The COVID - 19 pandemic significantly altered the educational landscape for adolescents, introducing new challenges to academic performance, particularly through the increased use of social media and rising levels of procrastination. Academic difficulties were amplified by the COVID - 19 pandemic, following the abrupt closure of schools and the shift to remote learning (104). The pandemic disrupted routines, increased stress levels, and created barriers to effective learning, particularly for students without access to adequate resources or those who struggled to adapt to online platforms (105). The lack of a structured learning environment at home contributed to a loss of academic performance that had lasting effects on adolescents’ mental health (104).

Low academic performance or repeated failures were strongly linked to an increased likelihood of suicidal ideation and attempts (31). Adequate school support and a positive school environment served as protective factors, reducing the risk of suicidal thoughts and self-harm (31, 36, 39, 106).

Adolescents facing academic challenges experienced intensified feelings of isolation and stress due to the lack of interaction and support (107). Improved access to digital resources, mental health interventions, and strategies to mitigate the psychological impact of academic stress could have been beneficial (104, 106).

Social media became a primary tool for adolescents to maintain connections during periods of isolation, but this also led to increased screen time, distracting students from academic tasks and promoting procrastination (108, 109). Long online engagement disrupted study routines and sleep schedules, which are critical for cognitive functioning and academic success (108, 110).

The shift to online learning intensified the temptation to engage with non-educational digital content, detracting from academic focus. Adolescents who struggled to regulate their social media use reported higher levels of academic stress and lower performance outcomes (108–110). The lack of in-person interactions with teachers and peers deprived students of essential support structures, undermining their ability to stay motivated and engaged with schoolwork (109). The interaction between procrastination and social media use during the pandemic reveals a cyclical relationship: adolescents turned to social media to avoid academic responsibilities, but this avoidance magnified feelings of guilt and anxiety about incomplete tasks, perpetuating a cycle of procrastination (108, 110).

Social isolation deprived adolescents of essential protective factors, such as meaningful peer interactions and structured environments, which typically buffer against psychological distress. For instance, the absence of in-person schooling not only limited access to mental health resources but also removed a critical outlet for problem-solving and stress management (110). This loss, compounded by family conflicts and economic instability, left adolescents with diminished opportunities to engage in constructive coping, forcing many to turn to harmful alternatives, including social media overuse and substance abuse to manage their distress (100, 109). Adolescents who previously relied on external support systems, including peers, teachers, and extracurricular activities, found these resources unavailable, leading to increased reliance on maladaptive behaviors such as avoidance, rumination, and self-harm (70, 71). Addressing these vulnerabilities requires a concerted effort to rebuild supportive school environments and strengthen interventions aimed at reducing academic stress, particularly in underserved populations (105, 106).

Lack of access to therapy, school counseling, and community programs significantly hindered adolescents’ ability to navigate heightened stressors. These barriers contributed to an increased prevalence of maladaptive behaviors, such as NSSI and suicidality, as adolescents struggled to process grief, fear, and uncertainty (96, 103). Additionally, the normalization of harmful behaviors through online platforms, such as exchanging dieting tips or self-harm methods, amplified these challenges (97, 101).

#### 4.2.5 Sociocultural and structural determinants as individual vulnerability factors

To enrich the cultural and geographic scope of the findings, data were incorporated from additional studies conducted in diverse global contexts, which provide further nuance to the understanding of adolescent suicidality and self-harm.

Community-based prevalence studies remain essential to understanding the hidden burden of adolescent self-harm. In a self-report survey conducted among adolescents aged 13 to 18 years in England, a lifetime prevalence of 12% for self-harm was observed, with higher rates among females and those aged 15-16. Importantly, over half of the adolescents who reported self-harming did not seek help, suggesting that official records may significantly underestimate the true scope of the problem. These findings underscore the need for proactive screening strategies within schools and community services to identify adolescents who do not access traditional clinical pathways (30).

Cultural factors and public stigma also influence the expression and reporting of self-injurious behaviors. In a South Korean study, differences were observed not only in prevalence by sex but also in the underlying motivations for self-harm. Girls were more likely to use self-injury as a coping mechanism for internalized emotional distress, whereas boys demonstrated more impulsive patterns. These distinctions highlight the importance of gender-sensitive prevention and treatment approaches, especially in non-Western contexts where cultural norms shape emotional expression and help-seeking behaviors (32).

The role of social connectedness and school environment was further emphasized in a multi-country study across ten Southeast Asian nations, which found that adolescents who reported loneliness, poor parental bonding, and low peer support were significantly more likely to engage in NSSI and experience suicidal ideation. In the context of heightened social isolation during the COVID - 19 pandemic, these findings draw attention to the urgent need for interventions that strengthen protective relationships at home and in school settings (33).

Emerging data from post-pandemic contexts suggest that adolescents experienced not only an increase in suicidal ideation but also a shift in the underlying factors contributing to such outcomes. In a recent analysis of adolescent suicide deaths in Maryland, it was observed that the most significant increases occurred among youth aged 10 to 14 years, particularly among males and black adolescents. The authors highlighted the importance of recognizing early developmental vulnerability and structural inequalities, which may have been exacerbated by the pandemic (34). Longitudinal evidence from van Vuuren et al. (45) further demonstrated temporal trends in suicidal ideation and attempts, with differences across sociodemographic groups. These findings support the notion that intersectional factors such as race, age, and socioeconomic context must be considered when analyzing temporal trends in youth suicidality.

Notably, studies conducted in Tanzania reinforced the association between teacher support, school engagement, and reduced suicidal ideation among adolescents, as well as the cumulative impact of psychosocial stressors such as bullying and family dysfunction (37, 38), mirroring patterns observed in diverse global contexts.

Taken together, these findings reinforce the urgent need for culturally sensitive, developmentally appropriate, and equity-driven strategies to address adolescent suicidality and self-harm across global settings. Integrating this broader lens into research, policy, and clinical practice is essential to build more inclusive and responsive mental health systems. As this systematic review highlights, understanding the changing dynamics of youth self-injury in the pandemic era demands not only epidemiological vigilance but also the centering of adolescent voices and local realities as a foundation for global action. Nonetheless, it is important to interpret these findings in light of several methodological and contextual limitations that may influence the generalizability and applicability of the conclusions.

### 4.3 Methodological considerations

The evidence base is constrained by the predominance of cross-sectional designs; non-probabilistic or school-based samples collected during periods of remote schooling and service disruption; heterogeneous outcome definitions and instruments; reliance on self-report; limited adjustment for confounders; and asynchronous data collection across different pandemic phases. These features introduce selection and measurement biases, restrict comparability, and limit temporal inference. Moreover, most included studies were conducted in North America, Europe, and East Asia, with comparatively fewer from Latin America and sub-Saharan Africa, reducing external validity to under-represented regions. Finally, potential publication bias and language bias due to English-only inclusion further limit generalizability. Accordingly, findings should be interpreted as a structured synthesis of patterns rather than precise pooled effect-size estimates, and causal inferences should be made with caution.

## 5 Limitations

This study faced several limitations that should be acknowledged. The diversity in methodologies, study designs, and population characteristics hindered the ability to integrate data effectively and draw consistent conclusions. The predominance of cross-sectional studies restricted the analysis to associations, without allowing for the establishment of causal links between risk factors and outcomes such as suicidality and NSSI. Additionally, the reliance on self-reported data introduced inherent biases, such as recall and social desirability biases, which may have compromised the accuracy of the reported behaviors and perceptions.

Moreover, this review retrieved no eligible studies from certain regions (e.g., Latin America, Francophone Africa), which may reflect publication bias, limited indexing in global databases, or lower research output from these regions. Such geographic gaps may limit the generalizability of findings and underscore the need for more inclusive, globally representative research efforts.

The adoption of digital tools for education and communication reshaped adolescent experiences, creating variability in risk factors and outcomes over time. Lastly, the reliance on digital platforms for data collection during the pandemic likely excluded adolescents from lower-income households with limited access to technology. This may result in an underestimate of the prevalence of mental health challenges among these groups, particularly in regions where digital differences were significant.

Despite these limitations, this study provides valuable insights into the complex interplay of risk factors influencing adolescent mental health during and beyond the pandemic. Future research should aim to address these gaps by adopting standardized methodologies, incorporating longitudinal designs, and ensuring inclusive sampling that captures the diverse and evolving experiences of adolescents worldwide.

## 6 Conclusion

The pre- and post-pandemic data analysis provides a nuanced answer to the question: {it}”Was there a change in the prevalence and risk factors of suicidality and self-harm among adolescents before and after the onset of the COVID - 19 pandemic up to the present day?”{/it} The answer is yes, with important distinctions. There is a significant rise in non-suicidal self-injury (NSSI) during the pandemic, while the prevalence of suicide attempts seems to decrease in pooled data. While the prevalence of suicide attempts decreased significantly during the pandemic (approximately 66.67%), NSSI exhibited a dramatic increase of about 992.86%.

Risk factors evolved and intensified throughout the pandemic change, with common challenges including lack of family support, academic difficulties, bullying, and mental health issues such as depression and anxiety, which persist to this day. In contrast, NSSI was strongly associated with poor emotional regulation, heightened loneliness, and the normalization of self-injury through online platforms. These findings highlight the distinct emotional and contextual pathways leading to these outcomes.

A complex interplay between pre-existing vulnerabilities and novel stressors, such as social isolation, economic hardship, and disruptions to routine particularly minority groups and poor faced disproportional severe impacts, emphasizing the interplay between socioeconomic and cultural dynamics in shaping mental health outcomes. Despite a decrease in suicide attempts, the persistence and escalation of NSSI behaviors highlight the enduring emotional toll of the pandemic, further exacerbated by restricted access to mental health services during critical periods. This represents a convergence of new and pre-existing challenges, carrying significant long-term repercussions.

Therefore, global mental health policy must now rise to this challenge with coordinated, equity-driven, and adolescent-informed responses that leave no voice unheard.

## Data Availability

The original contributions presented in the study are included in the article/**Supplementary Material** Further inquiries can be directed to the corresponding author.
