# Supplementary material for: Suicidality and self-harm in adolescents before and after the COVID-19 pandemic: a systematic review
Source: Front Psychiatry. 2025 Sep 19;16:1643145. doi: 10.3389/fpsyt.2025.1643145 (PMC12491249; doi:10.3389/fpsyt.2025.1643145)
Supplement: Supplementary file 1 [file DataSheet1.pdf]

Supplementary Material 1 – Table 1 – Syntax Table

The official search was conducted on 07/05/2024, combining the descriptors “adolescents”, “suicidality”, “self-harm”, and “prevalence”. For the post-pandemic search, the descriptor “COVID-19” was added. The descriptors were combined using the AND operator in the following databases: PubMed, PsycNet, Embase, and Scopus. To ensure a sensitive search, the eligibility criteria included: quantitative studies in community samples with populations aged 10–19 years (adolescents), published in English between 2010 and 2024.

Database	Syntax
PubMed	("adolescent"[MeSH Terms] OR adolescent*[Title/Abstract]) AND (suicid* OR "self-harm") AND prevalence [All Fields] Post-pandemic search: same strategy AND ("COVID-19"[MeSH Terms] OR COVID-19[Title/Abstract])
EMBASE	('adolescent'/exp OR adolescent*) AND ('suicidality'/exp OR 'self-harm'/exp) AND prevalence Post-pandemic search: same strategy AND 'COVID-19'/exp
PsycNet (APA PsycInfo)	Any Field: (adolescent* AND (suicid* OR "self-harm") AND prevalence) Post-pandemic search: add COVID-19
Scopus	TITLE-ABS-KEY (adolescent*) AND TITLE-ABS-KEY (suicid* OR "self-harm") AND TITLE-ABS-KEY (prevalence) Post-pandemic search: same strategy AND TITLE-ABS-KEY (COVID-19)
